# Women’s information needs around urine testing for urinary tract infections: a qualitative study

**DOI:** 10.3399/BJGP.2021.0564

**Published:** 2022-02-22

**Authors:** Margaret Glogowska, Caroline Croxson, Gail Hayward

**Affiliations:** Nuffield Department of Primary Care Health Sciences, University of Oxford, Oxford.; Nuffield Department of Primary Care Health Sciences, University of Oxford, Oxford.; Nuffield Department of Primary Care Health Sciences, University of Oxford, Oxford.

**Keywords:** Primary health care, general practice, urinary tract infections, qualitative research, urine specimen collection, bacterial infections

## Abstract

**Background:**

Urinary tract infection (UTI) is one of the commonest bacterial infections in general practice, with urine testing a frequent feature of its management. Urinary dipsticks are widely used, with urine culture the reference standard test. To avoid contamination, patients are advised to discard the first part of the urine stream, retaining the midstream part for the sample. This process, however, can be challenging both to explain and to perform. There is a lack of literature investigating women’s perceptions and understanding of urine sampling.

**Aim:**

To explore women’s understanding of urine collection, sample contamination, and how information from samples informed UTI management.

**Design and setting:**

Qualitative study embedded in a UK randomised controlled trial (RCT) of urinary collection devices (UCDs) among women attending primary care with a suspected UTI.

**Method:**

Semi-structured telephone interviews were conducted with 29 women participating in the RCT. Interviews were transcribed and thematically analysed.

**Results:**

Participants were not always aware about what midstream samples were and why they were preferable. They also lacked understanding about how urine samples may be contaminated, and sources of contamination. Participants experienced variability in the information received following analysis of their sample.

**Conclusion:**

Provision of clear information could help provide better urine samples, aiding the diagnosis of UTIs, presenting results with greater clarity, and creating less need for repeat samples. Sharing of information derived from uncontaminated samples may also support better UTI management, helping to reduce unnecessary prescribing and antibiotic resistance.

## INTRODUCTION

Urinary tract infection (UTI) is one of the commonest bacterial infections managed in general practice and accounts for 1%–3% of all GP consultations.^1^ UTI is more common in women, for whom the lifetime risk is 50% and annual incidence is estimated to be over 10%.^1^

Urinary dipsticks are widely used by clinicians to rule out UTI, and they are the most widely used near-patient test in primary care.^2^^,^^3^ The reference standard test is urine culture, the commonest microbiological investigation performed in the UK. National guidance recommends that urine culture is performed for all women presenting with microscopic haematuria and recurrent UTI in addition to all pregnant women.^4^ However, bacteria from the host’s skin and vaginal secretions can contaminate the urine sample resulting in a mixed growth or equivocal result. This is the case for around 30% of samples from women,^5^ and a repeat specimen is often required to be sent to guide care. To avoid contamination, patients receive advice to discard the early part of the stream of urine, which may contain the largest part of any contaminants, and to retain the midstream part of the sample to be sent for analysis.^6^ This process, however, can often prove challenging for healthcare professionals to explain and for patients to perform.

While the testing of urine samples is a common feature of diagnosis and treatment of UTI in primary care, there is debate in the literature about the place and clinical value of midstream urine collection. One systematic review of studies comparing urine sampling techniques did not find evidence to support the collection of midstream samples to improve accuracy of diagnosis of UTI.^7^ Hoelmkjaer *et al* in their primary study, however, found a difference in the accuracy of a point-of-care (POC) test when using a midstream sample compared to a first-void sample, and recommended midstream sampling in primary care when using POC testing.^8^ Similarly, there is lack of agreement about the value of midstream urine culture. A randomised controlled trial (RCT) comparing five different approaches to managing UTI found no benefit in the routine sending of midstream urine samples for laboratory testing to guide antibiotic prescribing compared with other management approaches (immediate antibiotics, delayed antibiotics, symptom score, and dipstick testing) on measures of symptom control, symptom duration, and re-consultation.^2^ However, urine culture has been argued to add value in ruling out UTI^9^ and to help determine antibiotic choices in recurrent UTI.^10^

Patient perception and understanding of urine sampling for infection has not been explored. To address this, a qualitative interview study was conducted with a subsample of the women with suspected UTIs who had enrolled in a three-arm RCT of two urinary collection devices (UCDs) (Whiz Midstream^11^ and Peezy^12^) and standardised verbal instructions reported in the *BJGP*.^13^ The primary aim was to gather information on their experiences and perceptions of using the UCDs. The broader aim — the focus of this article — was to explore more generally women’s understanding of urine collection, contamination of samples, and how information gained from samples informed the management of their UTIs.

**Table:** How this fits in

Urinary tract infection (UTI) is one of the commonest bacterial infections managed in general practice, with women being predominantly affected. Asking patients with suspected UTIs to produce midstream urine samples for testing, using urinary dipsticks and urinary culture, is a common feature of the management of UTIs in primary care. This qualitative study aimed to explore women’s understanding of urine collection, how contamination of urine samples can occur, and how analysis of their samples informed the management of their suspected UTI. The findings indicate that patients do not always have the understanding they need to help them produce uncontaminated urine samples and do not always receive information derived from urinalysis, which could reduce antibiotic consumption and antibiotic resistance.

## METHOD

### Design

A qualitative methodology was chosen to explore these issues, as this is an area where a directly pertinent literature is lacking. Qualitative research is highly appropriate for capturing and exploring people’s experiences and perceptions of phenomena, and semi-structured, individual interviews allow researchers to explore their selected topics but also allow participants to raise issues of significance to them.

### Sample

The RCT recruited women aged ≥18 years who presented to UK general practice with symptoms attributable to UTI, including at least one of dysuria, haematuria, or frequency of urination, and were able to give informed consent for participation. All RCT participants were asked whether they would be willing to be contacted about participating in an interview study. Of those who agreed, a purposive sample was selected ensuring a range of ages across a range of recruiting general practices. Women were given written information about the interview study and had the opportunity to ask questions. Those who consented were interviewed by telephone as soon as possible after completing their participation in the RCT. Informed consent was recorded verbally.

### Data collection

A topic guide was developed, based on available literature and expertise within the research team. Questions were included about using the UCDs, which were part of the RCT, but also explored the participant’s experiences around providing urine samples in general, their awareness of the nature and implications of contamination of samples, as well as their perceptions of how information derived from their samples might determine how their UTI was managed. The topic guide was used flexibly, allowed the interviewers to follow up issues raised by participants, and was modified as the study progressed (final version is included as Supplementary Appendix S1). The semi-structured individual interviews were conducted by two authors between December 2016 and February 2018, across the period when women were exiting the RCT. The interviewers — a health services researcher and a social sciences researcher — have longstanding experience in conducting qualitative interviews and have both taught qualitative research methods. Data collection ended when the researchers agreed that no new issues were emerging from the interviews and there was sufficient understanding of the emerging categories. The average duration of the interviews was 30 minutes.

### Analysis

The interviews were audiorecorded, transcribed verbatim by a transcription company, and transcripts returned to the researchers for checking and anonymisation. The data were analysed thematically, with the assistance of NVivo (version 11). Both researchers familiarised themselves with the transcripts and they collaborated in systematically coding the data and establishing a coding framework. They then moved on to exploring relationships between codes, which led to the development of categories (provisional groups of codes) and eventually themes, sharing and discussing these with the wider research team to ensure the their credibility and confirmability.^14^ The researchers employed a constant comparison strategy in the analysis process,^15^ moving between different parts of the dataset to check if ideas or categories developed in one part of the dataset were present in another part, and ensuring that all of the data were comprehensively explored. Feedback was not sought on the findings from the participants themselves but shared and discussed with the research team, which included Patient and Public Involvement representatives.

## RESULTS

Altogether, 29 women participated in the interviews; 26 had been randomised to one of the two devices and three to standard care in the RCT. Participants’ ages ranged from 20–88 years. Other characteristics of the sample are displayed in Table 1. When possible participants were contacted after they had exited the trial, three women declined to take part in the interview study.

**Table 1.** Participant characteristics

**Characteristic**	***n***
Recruited	29

**Age, years**	
20–29	5
30–39	2
40–49	4
50–59	4
60–69	4
70–79	8
80–89	2

**Previous history of UTI**	
Yes	24
No	5

**Antibiotics given for current UTI episode**	
Yes	27
No	2

*UTI = urinary tract infection.*

Analysis of the interviews revealed three themes reflecting areas where participants with a suspected UTI had information needs. These were awareness around the need to collect a midstream urine sample; awareness around how urine samples can be contaminated; and awareness around the information obtained from urine samples and what this means for antibiotic prescription.

Some of these information needs were expressed by participants themselves, some became evident to the interviewers as participants described their experiences of UTIs and were then further explored within the interview.

### Awareness around the need to collect a midstream urine collection

The sample contained women who did know about the need to produce midstream urine samples:

*‘I’ve been told that by doctors and the nurses when I’ve had to do samples, I’ve always been told, can you try and do a midstream please … so obviously you pee and then you do a midstream and then you finish off in the toilet.’*

(Interview 23, aged 73 years)

One participant was aware because of her work as a healthcare professional (HCP). Others were familiar because of being asked to provide samples during their pregnancies:

*‘I suppose it goes back to maternity days, I suppose, which is a long, long time ago.’*

(Interview 6, aged 70 years)

*‘So I think you get used to that if you’ve had a baby.’*

(Interview 28, aged 64 years)

While aware of the need for a midstream sample, some women questioned in practice how easy it was to produce one with a UTI, particularly with regard to the volume of urine necessary:

*‘If you haven’t got any and then you can’t do midstream because there is no midstream.’*

(Interview 1, aged 66 years)

*‘What they call midstream might actually be quite early on because they haven’t got much in them.’*

(Interview 19, aged 44 years)

or with a condition such as a prolapse of the womb:

*‘About half, yes about halfway through, this is what I was told to do and I mean more often than not I couldn’t do it … you’ve got to be able to stop peeing and if something’s sitting on your bladder you can’t do it.’*

(Interview 4, aged 76 years)

For a number of the participants, the information had been received more recently, having been explained to them in the course of taking part in the RCT. Other participants, however, did not mention being asked to produce a midstream sample and, when this was raised with them during the interviews, did not indicate that this was something they had tried to achieve.

One participant indicated that she had always lacked the information needed to produce a sample correctly and recommended more information be given by the HCPs involved:

*‘Maybe ... sort of information how to take your sample correctly because I haven’t been really aware of anything I can remember from my childhood like when taking urine samples … maybe just if nurses or just doctors sort of make sure repeat it, how to take the sample correctly, or maybe some information sheet, I don’t know.’*

(Interview 21, aged 73 years)

One participant recommended displaying information about collecting urine samples in GP surgeries to help with the issue:

*‘I think if it were advertised in the waiting room, as many things are, it would save a lot of women thinking, “How am I going to get that, am I going to use a cup or what am I going to use?” If it was displayed I think women would really value that.’*

(Interview 4, aged 76 years)

### Awareness around how urine samples can be contaminated

In a similar way, participants’ awareness and understanding about contamination of samples varied considerably.

#### Knowing that contamination can occur

The majority of participants were unaware of the possibility of contamination of urine samples:

*‘I didn’t know, I haven’t been aware of any possible contamination … that might be an issue with urine sample.’*

(Interview 21, aged 73 years)

or had been unaware until they participated in the RCT:

*‘I wasn’t aware of it beforehand but yeah, she* [practice nurse] *explained about how, you know, what the idea of the whole study was about, was to try and reduce the amount of samples that are contaminated.’*

(Interview 17, aged 22 years)

*‘I hadn’t been appreciating the fact about the contamination, I was really surprised about the document* [patient information sheet] *that I read and the last report that it was contaminated.’*

(Interview 29, aged 39 years)

Even participants who were aiming to produce a midstream sample did not necessarily link this to contamination:

*‘I’ve been told that by doctors and the nurses when I’ve had to do samples, I’ve always been told, can you try and do a midstream please … No. No, I haven’t, never been aware of that* [contamination of samples] *at all.’*

(Interview 23, aged 57 years)

By contrast, a small number of participants were aware that contamination of samples occurred:

*‘Yeah, I’ve read, I think it says on the label to … but it does say to do it midstream and stuff, so I am, I am aware that it can be contaminated.’*

(Interview 8, aged 27 years)

Two women attributed their awareness, not to their own experience, but to their professional background — one as a biologist and the other as an HCP:

*‘I’m doing a PhD in biology … but not from personal experience.’*

(Interview 14, aged 23 years)

*‘Yeah, I had been aware, I used to work as a midwife so I knew that happens.’*

(Interview 15, aged 42 years)

Other participants knew about contamination because of what they had been told or from their own experience of producing contaminated samples:

*‘I think occasionally there’s what they call faecal contamination.’*

(Interview 5, aged 74 years)

One participant who had been told she had provided contaminated samples would have also appreciated more information about how contamination occurred and how it could be reduced:

*‘I was at the same time a little bit disappointed because then my GP told me that … because of those contaminations she couldn’t told exactly what was going on, which was really disappointing for me … but maybe just if nurses or just doctors sort of make sure repeat it, how to take the sample correctly, or maybe some information sheet, I don’t know.’*

(Interview 21, aged 73 years)

#### Cause of contamination

When the topic of contamination of samples was raised in the interviews, only one participant saw the body as a source of contaminants as the sample was collected and queried whether an anti-bacterial wipe might help:

*‘But if it’s your urine, I mean where’s the cross-contamination coming from, is it concern that it’s coming from around the surrounding area or ...? Okay, so it’s nothing to do with the urine itself, it’s just what gets in there from ... but I’m wondering if that would be a way of like doing like an anti-bac wipe or something that’s not going to aggravate the person before you actually pee.’*

(Interview 11, aged 57 years)

Other women, who did think more generally about contamination of samples, only envisaged it as being to do with the cleanliness of the container they were using to collect the urine:

*‘I always thought, I didn’t like, I do have one, a pot in my cabinet in the bathroom and I always thought, oh well I would never use that because I don’t know if it’s really clean, you know, how can it still be clean?’*

(Interview 16, aged 53 years)

*‘I did know that* [about contamination] *but that’s why I use a very clean mug.’*

(Interview 18, aged 49 years)

### Awareness around the information obtained from urine samples and what this means for antibiotic prescription

A number of participants reported having their urine sample tested immediately using a dipstick. Some were informed that the dip showed infection or that blood was present:

*‘… just enough for her to know that there was an infection in there.’*

(Interview 9, aged 88 years)

*‘… he* [the GP] *then did the dipstick on that … I had blood … ‘*

(Interview 16, aged 53 years)

For one participant, receiving the information that an infection was present was very important as it guided her decision to take antibiotics, which she would otherwise have been very reluctant to do:

*‘... she dipped in the whatever it is, into the urine sample, and gave me a result straightaway, and that then meant that I was armed with sufficient information that then persuaded me that actually, for once in my life, I should take the antibiotics and feel much better on it.’*

(Interview 19, aged 44 years)

Ambivalence about taking antibiotics also made another participant glad to receive information from the urine samples, which ascertained the need for an antibiotic prescription:

*‘I’d rather they do that* [check the sample] *and then you know that it’s being checked or whatever, rather than just being given antibiotics, you know, and it might not be that … I’m always happy to do a sample and I’d rather do that to be honest with you … I’d rather know that you need them before taking them, you know, I’m, I don’t mind taking tablets but I don’t take them for the sake of it, do you know what I mean?'*

(Interview 26, aged 40 years)

Another participant also spoke about receiving information as a result of the urine dip and being prescribed an antibiotic. She also reported how her sample was usually sent away for analysis to check that she was prescribed the *‘right antibiotic’*:

*‘They test it straightaway to see if you have an infection or what traces of blood or whatever are in and they put the filter paper in I think or do something. And so then they send it off, if there is no trace of anything in it, but there always is when I go in with my sample … yeah, they send it off to get it analysed if there is something but in the meantime they will often give me an antibiotic to control, try and ... hoping they’ve got the right antibiotic.’*

(Interview 5, aged 74 years)

For other participants there was also a link between the testing of the urine sample and receiving the best antibiotic for the infection present:

*‘I was expecting to get my urine sample tested and to be given with like info, sort of pretty certain that I was, that I had an infection, the correct antibiotic for it.’*

(Interview 5, aged 74 years)

Where urine samples had been sent to the laboratory, some women were later told that they had received the right antibiotics or if they needed a different one:

*‘I don’t know, I didn’t really think much about it* [contamination of urine samples] *, you know, I just thought, well she* [the GP] *usually gives me an antibiotic, and then when I ring down about the results she says, “You were given the right antibiotics,” so and it clears up, so as far as I’m concerned that’s good enough.’*

(Interview 22, aged 73 years)

*‘And this time too actually, needed different antibiotics. Such a pain … I mean the first lot I wasn’t quite sure when I finished them, so I just took another sample in, and then actually I still had an infection so then just needed stronger antibiotics, different antibiotics.’*

(Interview 18, aged 49 years)

Other participants, however, were less certain about what information had been gained from testing their samples or felt conflicted about how the information guided the management of the suspected UTI:

*‘And it came back, it did come back and I got the results from the doctor … So he just said he was slightly confused about some of the symptoms and that’s why I went in recently to have a blood test and another urine sample … ‘*

(Interview 11, aged 57 years)

*‘*[the doctor] *said, well they’d tested these things, these samples and they hadn’t found a trace … but as she was confident that the urinary tract infection had now cleared I stopped taking it* [antibiotics] *… The only other thing … was that I found it slightly odd that they were talking about blood in the urine, mostly and not really about what type of bug they’d found and whether the antibiotic would be good for it or not.’*

(Interview 1, aged 66 years)

One participant explained that information from the urine dip had *‘ruled out’* a UTI but she remained convinced that this was what she had been experiencing:

*‘The only thing I had was that, because it didn’t culture, the nurse practitioner went, no you haven’t got one* [UTI] *… but the nurse practitioner in the GP surgery was like, no, we’ve had the results from your, the sample, hasn’t shown anything on the dip test, therefore you haven’t got one, so that was interesting … Yeah and the nurse was like, oh this means you haven’t one, and it was kind of like, well that’s not how it may be quite works, but … yeah and I didn’t hear anything afterwards, but they’d give me antibiotics and the symptoms went.’*

(Interview 14, aged 23 years)

Similarly for another participant the urine dip hadn’t shown anything and the rest of the sample was sent off for analysis:

*‘… she* [practice nurse] *did the dip test and I asked like was it fine and she said, yeah, it was fine but the nurse that I rang to get that appointment said that they should send it away and do the proper like more lab tests … yeah, when I went in they prescribed me antibiotics then but they did all the other tests and they all came back negative again so I apparently didn’t have a water infection.’*

(Interview 20, aged 20 years)

In both cases, antibiotics had still been prescribed. One other participant was unable to find out anything from urine samples taken either at the time of consultation:

*‘I mean I don’t think the doctor actually said to me, because he did test, he did test the wee but I don’t think he actually said to me, it was all a bit of a blur … so he didn’t actually say to me “yes, I can see you’ve got something” or “no you haven’t”, he just packed me off with the antibiotics.’*

(Interview 26, aged 40 years)

Or later from the laboratory analysis:

*‘I think the difficult thing is it’s getting the results back and knowing what come of it, do you know what I mean? You don’t always get that bit of information back … I don’t know what came of it, did they find something or not or, do you know what I mean? I think there’s that kind of it would just be nice to kind of really understand I guess.’*

(Interview 26, aged 40 years)

## DISCUSSION

### Summary

While participants were willing to provide urine samples during times of suspected UTIs, they were not always aware about midstream samples and why these are preferable. They also reported experiencing difficulty in providing midstream samples during a UTI, often due to quantity of urine. There are also gaps in their understanding about how urine samples may be contaminated, and the possible sources of contamination. Due to the difficulty in providing samples using the usual method of collecting urine in a small pot/tube, some women had devised their own methods to help, which may contribute to contamination. Addressing this knowledge gap around midstream samples and possible contamination could have the potential to result in improvements in the diagnosis and treatment of UTIs.

Among participants there was variability in the amount, quality, and timing of the information given to them about both dipstick and laboratory analysis of their urine samples. In few cases did the information appear to link up with whether antibiotic prescribing was necessary, whether alternative management strategies might be suitable, or how the antibiotic was appropriate for the infection present.

### Strengths and limitations

Qualitative interviews were the optimal method of data collection for this highly personal and relatively unexplored condition, and the interviews offer insights and useful perspectives on participant’s views and experiences. Interviewing participants by telephone may also have contributed to their speaking with frankness and apparent lack of embarrassment. Considerable variation in the sample was achieved, in terms of age, background, and geographical location across the UK.

Women who were interviewed had also agreed to take part in the RCT and to randomisation. For this reason, they may have already been more engaged with their health and sought better ways to manage conditions such as UTIs. This might suggest present findings may not be transferable to a wider general practice population.

### Comparison with existing literature

As with midstream urine sampling and urine culture, there is debate about the place of dipstick testing of urine samples in primary care. Public Health England (PHE) guidelines suggest that dipsticks can be used to rule out UTI,^4^ while the more recent guidance from the Scottish Intercollegiate Guidelines Network (SIGN) recommends the use of dipsticks to make diagnosis more accurate.^16^ It is noteworthy that both sets of guidelines do not recommend the use of dipsticks in women aged >65 years because of the likelihood of bacteria present in urine and the bladder without an infection. Yet a sizeable proportion of the women interviewed (*n* = 13/29) fell into this age category and some had experienced dipstick testing. Recently, dissatisfaction among clinicians has been expressed with both available tools for management of UTI — urine dipsticks and laboratory-based urine culture.^17^

While there is a considerable body of research around the contribution of testing of urine samples in diagnosing and managing UTI, the authors are not aware of any research specifically exploring women’s perceptions and understanding of it, and have found little evidence regarding women’s understanding of urine collection, contamination of samples, and how information the samples provide informs the management of UTIs. The present findings indicate that this is an area where the lack of understanding could impact on how samples are produced and consequently their usefulness in diagnosing UTI. To help them provide better samples, a number of participants explained how they would have welcomed verbal information from HCPs and written information in formats such as leaflets or on posters in their GPs’ surgeries.

Most women presenting with UTI symptoms to general practice are prescribed an antibiotic.^18^^–^20 However, empirical antibiotics are poorly targeted and GPs are challenged by decision making around when to prescribe antibiotics and, if required, the most appropriate choice of antibiotic.^17^ The present findings show that the information gained by participants from urine testing was variable. Some accounts from participants revealed that it was the evidence from the urine samples that convinced them that they should take antibiotics, which they would not otherwise have been keen to do. This relates well to findings in the literature that women may not necessarily be seeking antibiotics when they consult their GPs. There has been research into alternative approaches to the management of symptoms of suspected UTIs. Duane *et al* suggested that reduced antibiotic prescribing for UTI could be facilitated by dialogue between clinician and patient about antibiotics and other options for treatment.^18^ Leydon *et al* reported that some women found a delayed antibiotic prescription acceptable. This was particularly the case where they wished to avoid the negative effects of antibiotics and preferred a more ‘natural’ approach.^21^ Similarly there is other evidence that women are open to alternative management of suspected UTI and that they will accept a ‘watch and wait’ approach, adopting self-care strategies, such as drinking more fluids and cranberry juice, and taking over-the-counter preparations and symptomatic relief such as non-steroidal anti-inflammatory drugs.^22^^,^^23^

### Implications for research and practice

The present study findings indicate that women may not have the information they need to produce uncontaminated urine samples. It was found that women had been surprised to learn about the importance of midstream urine samples and the possibility of contamination. Their responses pointed to their willingness to try and improve the quality of the samples they produce for testing, had they been aware. Where women are required to produce urine samples, the provision of clear information could help to provide better samples that will aid diagnosis of UTIs, with greater clarity around results and less need for repeat samples. A patient information booklet, which includes practical information on why and how to collect a midstream urine sample, and how information from the sample can guide treatment, has recently been published by SIGN but this was not available at the time of the RCT or this qualitative study.^24^ Future research could be directed to the evaluation and further development of this and other materials, which could be supplied to women who present with suspected UTI.

The findings also provide some evidence that the sharing of information derived from uncontaminated samples may support better management of UTI. Women reported that information shared with them helped to reassure them about antibiotic prescribing decisions and gave them a better understanding about when antibiotics might or might not have been necessary. In turn, better sharing of information could contribute to the reduction of unnecessary prescribing and antibiotic resistance.
